# Telehealth Services Among Active Component Members of the U.S. Armed Forces, 2021-2025

**Published:** 2026-07-31

**Authors:** Adewumi Adegboye, Sithembile L. Mabila

**Affiliations:** Epidemiology and Analysis Branch, Armed Forces Health Surveillance Division, Public Health Directorate, Defense Health Agency, Silver Spring, MD: Mr. Adegboye, Dr. Mabila

Telehealth in the Military Health System (MHS) has long been an important tool for providing care in deployed and non-deployed settings. ^1^ The U.S. Department of War uses telehealth for primary care, medication management, ^2^ and other services including outpatient care. Certain types of care provided at fixed military hospitals and clinics, as well as health care encounters outside the military medical system that are billed to TRICARE, are also provided through telehealth. ^3^

This Surveillance Snapshot presents trends in telehealth service use and identifies the 10 most frequent diagnoses addressed via telehealth among U.S. active component service members (ACSMs) using Defense Medical Surveillance System (DMSS) outpatient and demographic records from January 2021 through December 2025. Telehealth services were identified by a virtual appointment type through video visit or My Military Health Virtual (MMH VIDEO) or by Common Procedural Terminology (CPT) codes 98966–98969, 99374–99380, 99339–99444, 99421–99423, 98000–98007, G0320–G0321, G0425–G0427, G0459, G0508–G0509, D9995, G2061–G2063, C7900–C7902, and T1014.

Use of telehealth was defined as having at least 1 telehealth encounter per patient per day; if a patient had multiple telehealth encounters per day, the first record was retained as the qualifying encounter. Reasons for telehealth encounters among ACSMs were determined using International Classification of Diseases, 10th Revision codes associated with each telehealth visit. The rate of tele-health encounters was calculated per 10,000 encounter records and stratified by year, patient demographics, and type of care (e.g., military hospitals and clinics or civilian care). Because the underlying database is static yet constantly updated, the quantitative figures presented in this report differ slightly from data reported for the same periods in prior analyses.

A total of 3,694,460 telehealth encounters were provided to over 1,147,951 ACSMs during the 5-year period. The overall unad-justed rate of telehealth per 10,000 encounters evinces an upward trajectory from 2021 through 2025, rising from 242.3 to 633.4 per 10,000 encounters **(Table)** . Over the 5-year period, women used telehealth at a higher overall rate than men (419.4 and 396.6 per 10,000 encounters, respectively), although this trend inverted in 2024 and 2025, with men availing telehealth services at higher rates.

**TABLE.** Rates of Telehealth Services ^a^ by Year, Active Component, U.S. Armed Forces, 2021–2025

Variable	Year					Total
	2021	2022	2023	2024	2025	
Overall rate	242.3	299.3	386.3	511.0	633.4	402.6
Sex						
Male	221.7	287.9	383.7	518.1	641.2	396.6
Female	302.0	330.7	393.4	491.5	612.9	419.4
Age, *y*						
<20	120.2	180.5	242.7	361.0	474.9	252.7
20–24	249.8	308.0	392.0	510.9	619.0	396.8
25–29	268.3	321.3	412.0	546.9	674.8	432.5
30–34	263.2	325.4	421.5	555.6	678.6	439.9
35–39	249.2	304.3	395.3	520.8	645.2	417.3
40	212.5	260.4	340.2	453.9	587.8	366.8
Race and ethnicity						
White, non-Hispanic	238.6	297.6	391.8	520.6	643.9	401.8
Black, non-Hispanic	243.9	278.4	352.3	467.5	587.5	376.8
Hispanic	245.1	309.5	385.2	502.4	622.6	408.7
Other	252.2	323.8	416.3	551.6	682.0	436.9
Branch of service						
Army	176.7	215.9	256.1	363.3	447.7	281.7
Navy	157.2	213.6	326.6	403.2	559.6	325.1
Air Force	486.6	514.6	626.0	862.2	1027.6	683.8
Marine Corps	144.5	257.3	387.8	476.7	625.1	359.8
Coast Guard	141.6	389.6	413.6	415.2	480.2	376.7
Space Force ^b^		683.0	1092.3	1194.2	1227.2	1161.0
Source of care						
Military hospital or clinic	302.0	399.0	559.8	758.4	953.2	559.1
Civilian care	13.6	11.5	10.5	9.1	4.9	9.7

a Unadjusted rates calculated per 10,000 medical encounters.

b Space Force data available starting in 2022.

The leading 10 reasons for telehealth encounters from 2021 through 2025 were other general symptoms and signs, obstructive sleep apnea, encounter for other administrative examinations, occupational Health Periodic Health Assessment examination, encounter for immunization, lower back pain, pain in right knee, pain in left knee, adjustment disorder with mixed anxiety and depressed mood, and pain in right shoulder (data not shown).

The highest rates were observed in 2025 among male service members (641.2 per 10,000 encounters), those ages 30-34 years (678.6 per 10,000 encounters), Space Force ACSMs (1,227.2 per 10,000 encounters), individuals treated by a military hospital or clinic (953.2 per 10,000 encounters), and ACSMs of ‘other’ races and ethnicities (682.0 per 10,000 encounters) **(Table)** .

The steady increase of telehealth encounter rates from 2021 through 2025 indicates a growing role for virtual care among ACSMs.
